# The Vestibular Drive for Balance Control Is Dependent on Multiple Sensory Cues of Gravity

**DOI:** 10.3389/fphys.2019.00476

**Published:** 2019-04-30

**Authors:** Anne I. Arntz, Daphne A. M. van der Putte, Zeb D. Jonker, Christopher M. Hauwert, Maarten A. Frens, Patrick A. Forbes

**Affiliations:** ^1^Department of Neuroscience, Erasmus MC, Erasmus University Medical Center, Rotterdam, Netherlands; ^2^Department of Biomechanical Engineering, Faculty of Mechanical, Maritime and Materials Engineering, Delft University of Technology, Delft, Netherlands; ^3^Department of Rehabilitation Medicine, Erasmus MC, Erasmus University Medical Center, Rotterdam, Netherlands; ^4^Rijndam Rehabilitation Centre, Rotterdam, Netherlands

**Keywords:** gravity, vestibular system, balance control, electrical vestibular stimulation, vestibular-evoked responses

## Abstract

Vestibular signals, which encode head movement in space as well as orientation relative to gravity, contribute to the ongoing muscle activity required to stand. The strength of this vestibular contribution changes with the presence and quality of sensory cues of balance. Here we investigate whether the vestibular drive for standing balance also depends on different sensory cues of gravity by examining vestibular-evoked muscle responses when independently varying load and gravity conditions. Standing subjects were braced by a backboard structure that limited whole-body sway to the sagittal plane while load and vestibular cues of gravity were manipulated by: (a) loading the body downward at 1.5 and 2 times body weight (i.e., load cues), and/or (b) exposing subjects to brief periods (20 s) of micro- (<0.05 g) and hyper-gravity (∼1.8 g) during parabolic flights (i.e., vestibular cues). A stochastic electrical vestibular stimulus (0–25 Hz) delivered during these tasks evoked a vestibular-error signal and corrective muscles responses that were used to assess the vestibular drive to standing balance. With additional load, the magnitude of the vestibular-evoked muscle responses progressively increased, however, when these responses were normalized by the ongoing muscle activity, they decreased and plateaued at 1.5 times body weight. This demonstrates that the increased muscle activity necessary to stand with additional load is accompanied a proportionally smaller increase in vestibular input. This reduction in the relative vestibular contribution to balance was also observed when we varied the vestibular cues of gravity, but only during an absence (<0.05 g) and not an excess (∼1.8 g) of gravity when compared to conditions with normal 1 g gravity signals and equivalent load signals. Despite these changes, vestibular-evoked responses were observed in all conditions, indicating that vestibular cues of balance contribute to upright standing even in the near absence of a vestibular signal of gravity (i.e., micro-gravity). Overall, these experiments provide evidence that both load and vestibular cues of gravity influence the vestibular signal processing for the control of standing balance.

## Introduction

Whenever we stand, the downward pull of gravity requires that we make continuous motor corrections to remain upright. Critical to this process is the ability to accurately estimate our orientation relative to gravity. The sensory cues that inform the brain about gravity, in the absence of vision, are derived primarily from the somatosensory and vestibular systems (see Dakin and Rosenberg, 2018 for a review). The somatosensory system encodes the gravitational load (i.e., forces) throughout the body within the local reference frame of the support surface, whereas the vestibular system’s otolith organs, together with the semicircular canal organs, encode head orientation within a fixed gravito-inertial reference frame. These sensory signals shape the corrective motor commands that maintain balance such that any change they undergo has an influence on the postural responses necessary to stand. For example, changes in body load (i.e., added or deducted weight) alters vestibular-evoked postural responses, whereby force rate production increases with loading and decreases with unloading (Marsden et al., 2003). Similar effects can also be observed when subjects take on asymmetric standing postures (Marsden et al., 2002), suggesting that load-related afferent feedback of gravity influences the processing of vestibular signals for the control of balance (Marsden et al., 2003).

Otolith-driven cues of gravity, in contrast to load-cues, appear to influence vestibulospinal reflexes only after prolonged (>24 h) exposures to changes in gravity. For example, in astronauts exposed to vertical drops on the first day of space flight, otolith-modulated motoneuron sensitivities (i.e., H-reflexes evoked during the drop) are analogous to levels tested pre-flight, but are almost absent after 7 days of space flight (Reschke et al., 1984, 1986). This change in otolith-driven motoneuron sensitivity suggests that the adaptation of vestibulospinal reflexes to changes in otolith cues of gravity may occur over longer periods than those observed for load cues of gravity during standing (Marsden et al., 2002, 2003). Vestibulospinal reflexes evoked by the drop conditions, however, arise from the otolith activity produced by the linear acceleration during freefall in absence of any requirement to stand. Therefore, these results may not be generalizable to the compensatory responses arising from the vestibular system during standing balance. Indeed, recent work has proposed that the characteristics of vestibular-evoked muscle corrections when standing reflect highly flexible responses centrally organized to compensate for vestibular disturbances (Britton et al., 1993; Fitzpatrick et al., 1994; Luu et al., 2012; Forbes et al., 2016). For instance, vestibular-induced lower-limb muscle responses during balance occur ∼30 ms later than those evoked by cortex stimulation (Britton et al., 1993; Dakin et al., 2016), even though the pathways conveying each signal have comparable conduction velocities. More notably, when standing subjects are restricted to balance in a single plane, vestibular-evoked muscle responses are greatest when the direction of a vestibular disturbance is aligned with the balance direction, and decrease to zero when the two directions become orthogonal (Forbes et al., 2016). This indicates that muscles compensate only for the component of the vestibular disturbance that is aligned with the balance direction, and not to the net vestibular activity that would be expected for vestibulospinal reflexes such as those produced during freefall. Therefore, we performed experiments to assess whether changes in otolith-driven signals of gravity – analogous to load cues of gravity – also modify the vestibular-evoked muscles responses for standing balance.

We used electrical vestibular stimulation (EVS) to evaluate the effects of varying load and vestibular cues of gravity on the corrective muscle responses required to stand. Electrical current applied to the mastoid processes distorts the firing rate of canal and otolith vestibular afferents (Kim and Curthoys, 2004; Kwan et al., 2019), and in a bilateral-bipolar configuration increases afferent activity on the side of the cathode and decreases afferent activity on the side of the anode (Goldberg et al., 1984; Kim et al., 2011; Kwan et al., 2019). The net sum of the afferent activity induces an isolated virtual signal of head rotation that is fixed in head coordinates (Fitzpatrick and Day, 2004; Day and Fitzpatrick, 2005; Peters et al., 2015) and is interpreted by the CNS as an unexpected vestibular disturbance. When standing, this vestibular disturbance evokes stereotypical muscle and whole-body postural corrections to maintain upright balance (Nashner and Wolfson, 1974; Lund and Broberg, 1983; Fitzpatrick et al., 1994; Dakin et al., 2007; Mian and Day, 2014; Forbes et al., 2016). Using this electrical stimulus, we performed experiments on ground and in parabolic flights to independently modulate load- and vestibular-related cues of gravity for balance. Load-related cues of gravity from the somatosensory system (i.e., cutaneous, proprioception) were modulated by loading subjects with 1.5 and 2 times their body weight using springs attached to the floor while subjects stood in 1 g gravitational conditions. Vestibular-related cues of gravity from the otolith end organs were then modulated by having subjects stand in micro-gravity (<0.05 g) and hyper-gravity (1.8 g) conditions while maintaining equivalent load cues via the springs. We found that the relative contribution of vestibular input to the corrective muscle responses was largest when subjects stood with normal 1 g related load and vestibular cues of gravity, and decreased when these cues were modulated. Specifically, with increased load cues of gravity, the relative vestibular-evoked responses decreased but remained constant when the load exceeded 1.5 times the body weight. Furthermore, responses decreased with modified vestibular cues of gravity, however, this effect was only observed in the absence (i.e., micro-g) and not the excess (i.e., hyper-g) of gravity. Despite these reductions, vestibular-evoked responses were observed in all conditions, indicating that vestibular contributions to balance are maintained even in the near absence of a vestibular signal of gravity (i.e., micro-gravity). Overall, these experiments provide evidence that both load and vestibular cues of gravity influence the vestibular signal processing for the control of standing balance.

## Materials and Methods

### Subjects

Twenty-one healthy subjects (*Experiment 1:* 16 subjects, mean age ± SD = 24 ± 4.2 years, mean height ± SD = 176 ± 7.1 cm, mean weight ± SD = 68 ± 7.7 kg, 10 men; *Experiment 2:* 6 subjects, mean age ± SD = 38 ± 8.3 years, mean height ± SD = 173 ± 9.4 cm, mean weight ± SD = 76 ± 9.7 kg, 5 men) with no known history of neurological disease or injury participated in this study. Subjects that participated in Experiment 2 completed both a training session under normal gravity conditions (*Experiment 2A*) and a flight session under variable gravity conditions (*Experiment 2B*) in the airplane. One subject participated in both Experiment 1 and Experiment 2. Experiment 1 was approved by the Medical Research Ethics Committee Erasmus MC and Experiment 2 by the University of Caen’s Ethics Committee. The experiments were conducted in accordance with the Declaration of Helsinki. All subjects gave their written informed consent prior to participation.

### Experimental Set-Up

**FIGURE 1:**
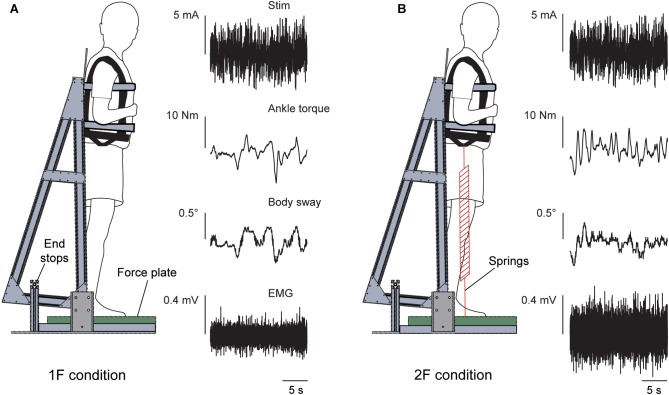
Experimental setup. The subject stood on a force plate and was strapped to a backboard setup that rotated in the sagittal plane about an axis that passed through the subject’s ankles. End stops functioned as angular limits to prevent the subject and backboard system from falling forward or backward. The subject remained upright in a slightly forward whole-body sway angle with normal 1 g body load or with added load. Raw data of the vestibular stimulus, ankle torque, whole-body sway angle and EMG activity of the right medial gastrocnemius are shown during a trial of Experiment 1 in the 1F **(A)** and 2F conditions **(B)**.

Two separate experiments were performed to study the effects of load and vestibular cues of gravity on the vestibular-evoked muscle responses. Experiment 1 assessed the influence of load cues of gravity under a constant gravitational level of 1 g. Experiment 2 assessed the influence of vestibular (otolith) cues of gravity under constant load levels of ∼1 and 2 times body weight. For both experiments, subjects maintained upright balance while being exposed to the stochastic EVS signal (see “Vestibular Stimuli”). In both experiments, subjects stood barefoot on a force plate (BP400600HF; AMTI, Watertown, MA, United States) with their feet 5 cm apart and their body secured to a backboard structure positioned immediately behind them (Figure 1). The backboard structure was used to eliminate any stabilizing effect of the subject loading system in the mediolateral direction (i.e., the downward pull of the springs, see below). The weight of the backboard structure was 10 kg with the center of mass at a height of ∼0.7 m. The backboard structure was supported by two bearings, such that the mass of the backboard only increased the subjects’ inertia by ∼6.5%. The backboard’s axis of rotation was fixed at a height of 7.5 cm above the top surface of the force plate and passed through the approximate location of the ankle joints (Huryn et al., 2010). As a result, whole-body sway was limited to the sagittal plane only. This pivoting direction corresponds with the direction of EVS-evoked whole-body sway responses when the head is turned over the shoulder (Lund and Broberg, 1983; Britton et al., 1993; Fitzpatrick et al., 1994). Angular limits of 10° anterior and 6° posterior from vertical prevented the subjects from falling forward or backward, respectively. Seatbelts across the chest and waist secured the subjects to the backboard. A laser distance sensor (optoNCDT-1401; Micro-Epsilon, Orteburg, Germany) attached to the backboard was used to record whole-body sway angle.

To control the vertical loading forces under varying gravitational levels (see section “Experiment 1” and “Experiment 2”), subjects wore a subject loading system to provide additional vertical load. The subject loading system consisted of a body-harness (German Aerospace Center (DLR), Cologne, Germany) and four springs. The body-harness was secured over the subject’s shoulders and tightened at the waist. The springs were attached to both sides (two springs on each side) of the body-harness using straps located at the height of the hips (i.e., at the subject’s approximate center of mass) and to a low-friction rail-trolley system secured to the floor. This rail-trolley system ensured that ground attachment of the springs moved with the center of mass of the subject such that the springs were always pulling vertically downward. This way, the intrinsic dynamics of the subject (i.e., the load-stiffness relationship) would match conditions appropriate for each load and gravitational level.

### Vestibular Stimuli

Stochastic electrical vestibular stimulation was delivered to the subjects in a bilateral bipolar electrode configuration via carbon rubber electrodes (∼15 cm^2^). The electrodes were coated with Spectra360 electrode gel (Parker Laboratories, Fairfield, NJ, United States) and secured over the mastoid processes with tape and an elastic headband. The skin over the mastoid processes was anesthetized with Pliaglis cream [lidocaine and tetracaine] (Galderma, Lausanne, Switzerland) to minimize cutaneous sensations under the electrodes. The stimuli were generated on a laptop with custom MATLAB software (MathWorks, Natick, MA, United States) and were sent to an isolated bipolar current stimulator (DS5; Digitimer, Hertfordshire, United Kingdom) via a data acquisition board (USB-6259; National Instruments, Austin, TX, United States). For both experiments, the electrical stimuli were designed as bandwidth limited stochastic signals (0–25 Hz, zero-mean low-pass filtered white noise, 25 Hz cutoff, zero lag, third-order Butterworth) with a peak amplitude of 5 mA [root mean square (RMS) of ∼1.7 mA]. The frequencies (0–25 Hz) contained in our electrical stimuli capture the entire bandwidth of vestibular-evoked muscle activity contributing to postural corrections (Dakin et al., 2007, 2010, 2011). This allowed us to provide a detailed assessment of any changes in vestibular contributions across conditions. In Experiment 1, a stimulus of 40 s was repeated four times in each condition (see section “Experimental protocol”; “Experiment 1”). In Experiment 2, a stimulus of 20 s was used to fit within the different gravitational phases of the parabola (see section “Experimental protocol”; “Experiment 2”) and repeated seven or eight times in each condition.

### Experimental Protocol

Prior to each experiment, a target whole-body sway angle was defined for each subject. This angle was 3° forward from their subjective zero angle; i.e., the position that subjects perceived as requiring minimal effort to stand upright. For each trial, subjects were instructed to stand upright, lean forward to their target sway angle, cross their arms over their chest, and rotate their head axially to the left (i.e., leftward yaw). The head was also rotated in extension such that the Reid plane was tilted up by 18° horizontally. This head position maximizes the postural responses to binaural bipolar EVS in the anterior-posterior direction along the line of action of the right medial gastrocnemius and soleus muscles due to the well-established orientation of the EVS vector (Lund and Broberg, 1983; Fitzpatrick and Day, 2004; Cathers et al., 2005; Day and Fitzpatrick, 2005) produced by the activation of all vestibular afferents (Kwan et al., 2019). Symmetry of otolith afferents across the striola of the utricle (Tribukait and Rosenhall, 2001) is estimated to result in the cancelation of an otolithic signal during electrical stimulation and a net EVS-vector that predominantly reflects canal activation (i.e., rotation) (Fitzpatrick and Day, 2004). To guide the subjects to their appropriate head and body position before each trial, they were given a subject-specific visual target that was placed on the wall to their left. In Experiment 1, a laser pointer attached to the subject’s head was used to orient the head in the desired position. In Experiment 2, the subject was instructed to align their head visually by looking at a target placed ∼1.5 m away on the aircraft wall since for safety reasons a head mounted laser could not be used in the aircraft. Subjects closed their eyes throughout each trial and were given verbal feedback regarding the whole-body sway angle and head position to help maintain a similar position over all trials.

#### Experiment 1

Experiment 1 assessed three different load conditions to examine the influence of load cues on the vestibular control of balance. Subjects stood with cumulative load forces through the feet equivalent to 1, 1.5 and 2 times their own body weight (conditions 1F, 1.5F and 2F, respectively) by progressively increasing the tension in the springs of the subject loading system. For each condition (1F, 1.5F, and 2F), subjects completed four 40-second trials (12 trials total) providing a total of 160 s of data for analysis under each condition. The order of the trials was randomized for each subject. Prior to delivering the electrical stimulus, subjects were instructed to lean forward to their offset angle, point the head-mounted laser to the mark and close their eyes. Experiment 1 was performed in the Department of Neuroscience at Erasmus Medical Center.

#### Experiment 2

Experiment 2 was performed with six subjects during the 68th ESA Parabolic Flight Campaign in a modified A310 Zero-G airplane (Novespace, Bordeaux, France) and consisted of a training session (Experiment 2A) and a flight session (Experiment 2B). The training session was completed on-ground in the aircraft 1 day prior to each subject’s participation in a parabolic flight (i.e., Experiment 2B). The training session familiarized the subjects with the experimental protocol and provided base-line data for qualitative comparison to Experiment 1 and Experiment 2B. The experiment was performed under two different loading conditions – 1F and 2F – following a similar protocol as described for Experiment 1. A 20-second electrical stimulus was used for Experiment 2A (and Experiment 2B), resulting in eight trials for each loading condition per subject. The order of trial condition (1F and 2F loading) was randomized for each subject.

**FIGURE 2:**
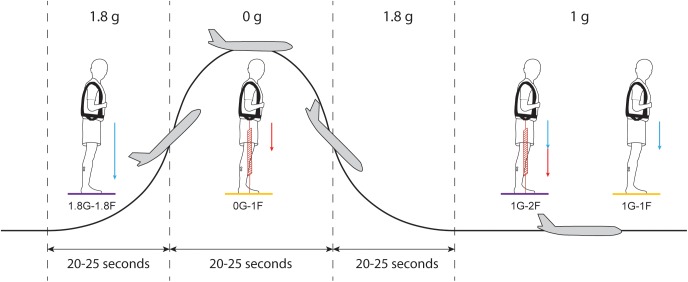
Protocol of Experiment 2B. Each parabolic maneuver starts with a hyper-g phase that is followed by a micro-g phase and ends with a second hyper-g phase. In between each parabola, there is a ∼100 s period of steady flight. For each gravitational phase, subjects performed standing balance tasks under different spring loading conditions while being exposed to the electrical vestibular stimulus. Blue arrows represent the load induced by gravity and red arrows represent the load induced by the subject loading system. Statistical comparisons were made between the results of the 0G-1F and 1G-1F conditions (yellow) and between the results of the 1.8G-1.8F and 1G-2F conditions (purple).

During the in-flight session, the airplane carried out parabolic flight maneuvers (Figure 2) that produced periods of weightlessness (i.e., micro-g or ∼0 g) and increased gravity (i.e., hyper-g or 1.8 g), which modified the vestibular cues of gravity. Each parabola started and ended with hyper-g periods of ∼20–25 s, separated by a ∼20–25 s micro-g period. Between each parabola the plane was in steady-flight (i.e., normal-g or 1 g) for approximately 100 s. The onset of the electrical stimulus was automatically triggered by acceleration along the *z*-axis of the plane (i.e., the gravitational loading direction). For the micro-gravity (micro-g) phase, the stimulus was triggered 2 s after *z*-acceleration fell below 0.2 g, and for the hyper-gravity (hyper-g) phase, the stimulus was triggered 2 s after z-acceleration exceeded 1.5 g. In the normal-gravity (normal-g) phase, stimulation was started 20 s after the second hyper-g phase of the parabola ended, i.e., when z-acceleration fell below 1.2 g. Offline examination of acceleration data confirmed that the 20 s stimulus occurred within the specific gravity phase for all trials.

Subjects participated in the experiment for 15 parabolic maneuvers (Figure 2) under four different conditions with varying levels of gravity (i.e., 0G, 1G or 1.8G) and load via the springs (i.e., 1F or 2F). During seven parabolas, subjects performed normal-gravity/normal-load trials (i.e., 1G-1F) and hyper-gravity/additional-load trials (i.e., 1.8G-1.8F) without spring loading in the normal-g and hyper-g phases, respectively. Hyper-gravity trials (i.e., 1.8G-1.8F) were performed during the first hyper-g phase of the parabola since a more consistent gravity could be achieved during this phase of the parabola. During the other eight parabolas, subjects performed the micro-gravity/normal-load trials (i.e., 0G-1F) and normal-gravity/additional-load trials (i.e., 1G-2F) with spring loading in the micro-g and normal-g phase, respectively (Figure 2). The order of the two groups of parabolas (with and without spring loading) was counter balanced across subjects. The subject load system was set per subject to exert a constant force equal to their own weight. Due to the strict timing of consecutive parabolic phases, we were unable to adjust the subject loading system such that the loading level in a steady flight (i.e., 1G-2F) was exactly the same as the load level during the hyper-g phase (i.e., 1.8G-1.8F). Therefore, comparisons of the vestibular-evoked responses were made between 0G-1F and 1G-1F trials, where foot loading forces were matched, and between 1.8G-1.8F and 1G-2F trials, where foot loading forces differed slightly (see Figure 2). These comparisons allowed us to evaluate whether changes in gravity-driven otolith signals (i.e., 1 vs. 0 g, and 1 vs. 1.8 g) influence the vestibular control of balance, while maintaining approximately equal load-related afferent cues.

During Experiment 2B, unexpected plane accelerations due to turbulence caused some subjects to fall into the backboard end stops in the middle of a trial. When this occurred, the trial was removed from further analysis. In addition, two subjects experienced motion sickness during the flight and skipped 1–3 parabolas. Nevertheless, all subjects performed a minimum of four trials (i.e., 80 s) per condition without falling into the end stops. For subjects who performed more than four trials without falling into the end stops, the four trials with the lowest mean variability of whole-body sway angle per condition were used for data analysis. This was necessary to maintain equivalent significance thresholds for all subject data (see section “Signal Analysis”).

### Data Recordings

In all experiments, surface EMG was collected from the medial gastrocnemius (mGAS) and soleus (SOL) muscles in the right leg using self-adhesive Ag-AgCl surface electrodes (BlueSensor M; Ambu, Copenhagen, Denmark). The recordings were made using a bipolar set-up with electrodes placed in-line with the muscle fibers at an inter-electrode (i.e., center-to-center) distance of 18 mm. The skin of the subject’s right leg was shaved and cleaned with skin preparation gel (NuPrep; Weaver and Company, Aurora, CO) and alcohol (MediSwab; BSN Medical, Hamburg, Germany) before the electrodes were secured. Acceleration of the plane was measured with a 3-axis accelerometer (3D Accelerometer; TMSi, Oldenzaal, Netherlands) and together with EMG was digitized at 2000 Hz on a data acquisition board (Porti7; TMSi, Oldenzaal, Netherlands). Vestibular stimuli, force plate signals and laser sensor data were digitized at 2000 Hz and recorded via a separate data acquisition board (USB-6259; National Instruments) using a custom MATLAB script (MathWorks, Natick, MA, United States). The two recording systems received a trigger signal at the onset of the vestibular stimulus to synchronize the data.

### Signal Analysis

Digitized EMG was high pass filtered offline using a zero-lag sixth order Butterworth filter with a cut-off frequency of 30 Hz and full-wave rectified. EMG signals for each trial were time-locked to EVS onset using the shared trigger signal. Data were concatenated per condition per subject, producing a single 160 (Experiments 1 and 2A) or 80 s (Experiment 2B) data array for a subject’s responses for each condition. Data from all subjects were then concatenated to create a single 2560 (Experiments 1 and 2A) and 480 s (Experiment 2B) pooled data set for each condition. Coherence and cumulant density functions were calculated with the individual and pooled data from each condition to evaluate the correlation between the electrical stimulus input and the rectified EMG of the two muscles (Dakin et al., 2007, 2010; Forbes et al., 2014). Data from all experiments were cut into 1 s segments, yielding a frequency resolution of 1 Hz, before computing the auto-spectra and cross-spectrum for the EVS and EMG data.

Coherence provides a measure of the linear relationship between the electrical stimulus (i.e., input) and rectified EMG (i.e., output) across a given range of frequencies. At each frequency point, coherence varies between 0 (no linear relation) and 1 (a linear relation with no noise). Coherence was defined as significant when exceeding the 95% confidence limit, as derived from the number of disjoint segments (Halliday et al., 1995). Coherence was estimated for each condition with concatenated data within each participant as well as concatenated pooled data for each condition across all subjects (see section “Statistics” below). Individual-subject coherence estimates were used to ensure responses were exceeding significance and consistent with the pooled data. The absence of significant coherence at all frequencies between the input stimulus and output muscle activity would indicate the suppression of vestibular contributions to balance.

Cumulant density functions provide a time domain measure of the relationship (i.e., cross-covariance) between the stochastic signal and the muscle responses and were calculated by taking the inverse Fourier transform of the cross-spectra (Halliday et al., 1995). Cumulant densities of individual-subject data are used throughout this study to assess the magnitude of the vestibular-evoked muscle response. To account for differences in EMG level between conditions, the cumulant density responses were normalized (between −1 and +1) by the product of the vector norms of the EVS input signal and EMG output signal (Dakin et al., 2010), allowing for inter- and intra-subject comparisons by minimizing potential bias induced by changes in EMG activity. Although this normalized cumulant density is more commonly used to evaluate changes in vestibular-evoked responses (Dakin et al., 2010; Reynolds, 2010; Dalton et al., 2014; Forbes et al., 2014, 2016), we also examined the non-normalized cumulant density responses to assess whether any changes in the normalized responses were modulated simply because of increased non-vestibular input to the motoneuron pool at higher load levels where muscle activity is expected to increase. Because this additional measure is not normalized by ongoing muscle activity, it is not expected to change between conditions if only non-vestibular input (e.g., corticospinal, reticulospinal, spinal reflexes, etc.) leads to increasing EMG magnitude. In contrast, a proportional increase (or decrease) in both vestibular and non-vestibular input is expected to increase (or decrease) only the non-normalized response. Therefore, by comparing the normalized and non-normalized cumulant density responses together with EMG magnitudes, we estimated how the relative vestibular contribution co-varies with the net input to motoneurons. In lower limb muscles, both the normalized and non-normalized cumulant density function exhibit a typical biphasic pattern with peaks defined as short (50–70 ms) and medium (100–120 ms) latency and occurring in opposing directions (Nashner and Wolfson, 1974; Britton et al., 1993; Fitzpatrick et al., 1994; Fitzpatrick and Day, 2004; Dakin et al., 2007, 2011). For comparison across conditions, the peak-to-peak amplitude of the normalized and non-normalized cumulant density was extracted from each subject’s response. We also extracted the timing of the peaks for each subject since changes in body load are known to modify the rate of vestibular-evoked postural responses (Marsden et al., 2003). Changes in the timing of the peaks could indicate a slower or more rapid development of a vestibular-evoked postural response. Finally, we estimated the ankle torque generated by subjects using the measured forces and moments, and the anatomical location of the ankle relative to the force-plate surface (Luu et al., 2011).

### Statistics

Because most subjects had never balanced under conditions with altered load and vestibular cues of gravity, we first evaluated changes in general balance behavior across conditions, including RMS muscle activity, vertical loading forces, estimated ankle torque, and whole-body sway angle (mean and mean-removed RMS). Analyses of these measures from Experiment 1 showed that the data were normally distributed, therefore the effect of load was identified using a repeated-measures ANOVA (Experiment 1: 1F/1.5F/2F). However, given the low subject numbers in Experiment 2A and 2B, for these data we used a Wilcoxon signed rank test to compare general balance behaviors across conditions (Experiment 2A: 1F vs. 2F; Experiment 2B: 1G-1F vs. 0G-1F and 1G-2F vs. 1.8G-1.8F).

To test the hypothesis that sensory cues of gravity modify the vestibular control of balance, we then examined the pooled coherence, the amplitude of the normalized and non-normalized cumulant density responses and the timing of the normalized cumulant density response across our various experimental conditions. We first evaluated the effect of load cues of gravity in Experiment 1 (i.e., 1F vs. 1.5F vs. 2F) on the pooled coherence using a difference of coherence (DoC) test. The DoC test was applied on the Fisher transform (tanh^−1^) of the coherency (square root of the coherence) values and compared to a χ^2^-distribution with *k* - 1 degrees of freedom (*k* is the number of conditions included in the comparison; *k* = 2). We then evaluated the effect of load cues of gravity (i.e., 1F vs. 1.5F vs. 2F) on the normalized and non-normalized cumulant density responses using a Friedman test. We used a non-parametric test because the peak-to-peak amplitudes and peak timing of the cumulant density responses were not normally distributed. When significant differences were observed, we performed pairwise comparisons (Wilcoxon signed rank test, Bonferroni corrected) to decompose the main effect of load across our three conditions. We also assessed whether a comparable trend in responses was observed in subjects participating in the parabolic flights by comparing responses across the lowest and highest load conditions in Experiment 2A (i.e., 1F vs. 2F) using a DoC test on the pooled coherence and a Wilcoxon signed-rank test on the cumulant density responses. Finally, we evaluated the effect of vestibular cues of gravity on the vestibular-evoked responses in Experiment 2B – 1g vs. 0g under normal load (i.e., 1G-1F vs. 0G-1F), and 1g vs. 1.8 g under additional load (i.e., 1G-2F vs. 1.8G-1.8F) – using a DoC test on the pooled coherence and Wilcoxon signed-rank tests on the cumulant density responses. All normal data are expressed as means ± standard deviations (SD) and non-normal data are expressed as medians and interquartile ranges (IQR). For all tests, statistical significance was set at 0.05.

## Results

### Effect of Load Cues of Gravity on Vestibular-Evoked Muscle Responses (Experiments 1 and 2A)

**FIGURE 3:**
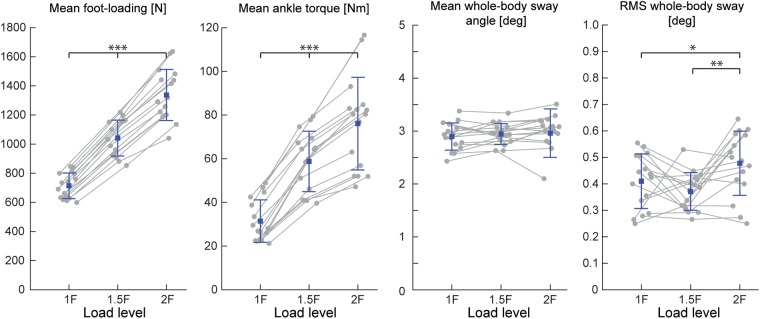
Outcome measures of general balance behavior during Experiment 1 (*n* = 16). Responses include vertical loading forces, ankle torque and whole-body sway angle (mean and mean-removed RMS). Individual subjects are plotted as gray dots. Group responses are plotted with means (blue dots) and standard deviations (whiskers). ^∗^*p* < 0.05, ^∗∗^*p* < 0.01, and ^∗∗∗^*p* < 0.001 indicates significant differences between conditions. mGAS = medial gastrocnemius muscle, SOL = soleus muscle.

**FIGURE 4:**
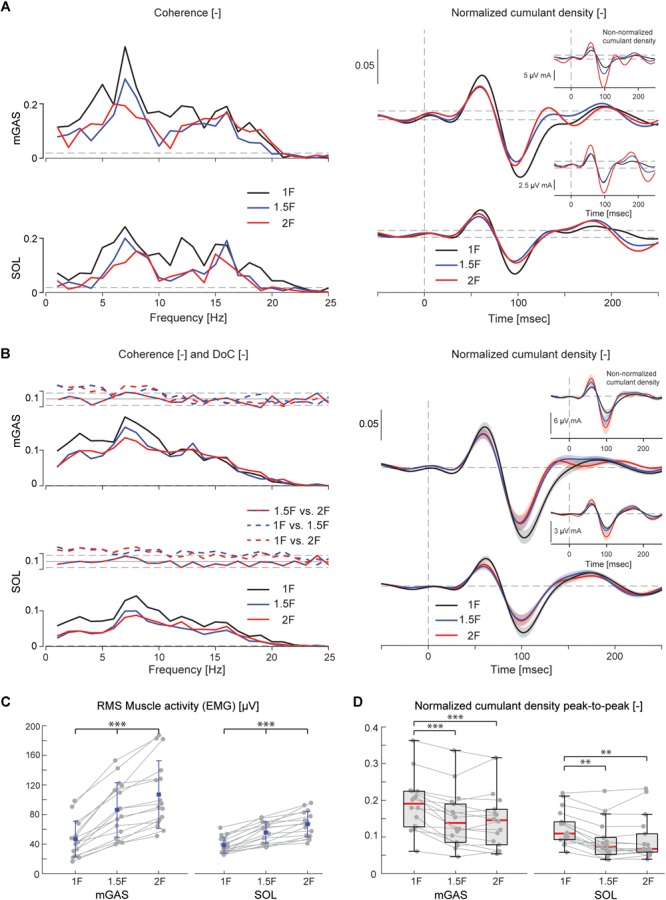
Vestibular-evoked muscle responses with varying load levels from Experiment 1. Data from the medial gastrocnemius and soleus muscles are shown for a single subject **(A)** and group responses (*n* = 16) **(B)**. Horizontal dotted lines indicate the 95% confidence limits for coherence, difference-of-coherence (DoC) and cumulant density responses. DoC results are plotted above the group pooled coherence results. A positive value for the DoC indicates greater coherence for the first condition listed in each comparison; whereas a negative value represents greater coherence for the second condition in each comparison. Cumulant density plots show the normalized and non-normalized (insets) responses for both individual subject and group data. In the group cumulant density responses, bold lines are group means and for illustrative purposes shaded areas show the standard error. For comparison, data from the group (*n* = 16) root-mean-square of muscle activity (EMG) **(C)** and peak-to-peak amplitudes of the normalized cumulant density responses **(D)** are shown. Individual subjects are plotted as gray dots. Group responses for normally distributed data are plotted with means (blue dots) and standard deviations (whiskers), while non-normally distributed data are plotted with medians (red line), 25 and 75 percentiles (gray box) and extreme data points (whiskers). ^∗∗^*p* < 0.01 and ^∗∗∗^*p* < 0.001 indicates significant differences between conditions. mGAS = medial gastrocnemius muscle, SOL = soleus muscle.

During Experiments 1 and 2A, all subjects were able to balance themselves at the identified target angle (see Table 1) in all loading conditions without difficulty. During Experiment 1, mean whole-body sway angle was comparable across the 1F, 1.5F and 2F loading conditions [*F*_(1.37,20.59)_ = 0.411, *p* = 0.592], however, the mean removed RMS sway angle varied depending on the specific load [*F*_(2,30)_ = 7.650, *p* = 0.002] (see Figure 3). Pairwise comparisons revealed that the highest load (2F) increased RMS sway compared to 1F and 1.5F conditions (*p* = 0.036 and *p* = 0.006, respectively), whereas there was no difference between the two lower load conditions (*p* = 0.659). As expected, the additional load during 1.5F and 2F conditions required increased medial gastrocnemius [84.4 and 128.6%, respectively, *F*_(2,30)_ = 77.3, *p* < 0.001] and soleus muscle activity [44.6 and 73.6%, respectively, *F*_(2,30)_ = 69.083, *p* < 0.001] relative to the 1F condition (see Figure 4C), as well as ankle increased torque [86.9 and 142.0%, respectively, *F*_(1.21,18.08)_ = 156.1, *p* < 0.001] (see Figure 3). Mean vertical loading forces in the 1.5F and 2F conditions were slightly below (145.9 and 187.3%, respectively) the intended load levels of 1.5 and 2 times body weight. This was likely due to a downward shift of the subject loading system over the pelvis throughout the trials, which reduced the load applied by the springs.

**Table 1:** Group data for measures of general balance behavior.

		RMS EMG		Foot-loading	Ankle torque	Sway angle	RMS angle
		mGAS [μV]	SOL [μV]	[N]	[Nm]	[°]	[°]
		*mean ± SD*	*mean ± SD*	*mean ± SD*	*mean ± SD*	*mean ± SD*	*mean ± SD*
**Exp 1(*n* = 16)**	*1F*	46.79 ± 24.16	38.54 ± 10.82	714 ± 88	31.44 ± 9.71	2.90 ± 0.26	0.41 ± 0.10
	*1.5F*	86.28 ± 36.78	55.74 ± 14.31	1042 ± 123	58.77 ± 13.82	2.94 ± 0.20	0.37 ± 0.07
	*2F*	106.97 ± 45.78	66.90 ± 17.01	1337 ± 176	76.08 ± 21.22	2.96 ± 0.46	0.48 ± 0.12

		***median/IQR***	***median/IQR***	***median/ IQR***	***median/IQR***	***median/IQR***	***median/ IQR***

**Exp 2A(*n* = 6)**	*1F*	53.33/23.09	40.27/15.31	817/118	40.06/13.69	2.96/0.09	0.58/0.19
	*2F*	89.64/31.31	69.11/19.18	1517/243	85.96/24.78	3.05/0.05	0.47/0.14
**Exp 2B(*n* = 6)**	*0G-1F*	39.79/28.28	43.57/12.56	647/166	36.91/16.51	2.73/0.99	1.28/0.57
	*1G-1F*	64.85/31.09	50.17/9.86	795/101	51.96/18.07	2.07/0.62	1.18/0.81
	*1.8G-1.8F*	118.28/34.99	98.87/16.94	1325/179	85.01/17.29	-2.00/0.67	3.08/ 0.92
	*1G-2F*	92.91/37.28	72.26/12.66	1405/259	85.21/30.15	2.19/0.46	0.70/0.41

*For normally distributed data, mean and standard deviation (SD) are given, while for non-normally distributed data, median and interquartile range (IQR) are given. mGAS, medial gastrocnemius muscle; SOL, soleus muscle.*

Lower limb muscle activity showed significant correlation with the electrical stimulus for all subjects and in all conditions. Data from a representative subject show that coherence in both muscles was significant at frequencies up to about 20 Hz (Figure 4A). The associated biphasic muscle response (i.e., normalized cumulant density) produced short-latency (∼70 ms) and medium-latency (∼100 ms) peaks exceeding the 95% confidence interval (Figure 4A). With increasing load (1.5F and 2F), the coherence and the normalized cumulant density responses decreased by similar amounts across the two conditions, while the non-normalized cumulant density progressively increased with load (see Figure 4A insets). A similar trend was observed in the group data (see Figure 4B): the DoC test revealed a significant decrease in pooled coherence in both muscles between 0 and ∼10 Hz when the load was elevated (1.5F and 2F), however, between the two load conditions (1.5F vs. 2F) there was no change in coherence. This was associated with a significant effect of load condition on the peak-to-peak amplitude of the normalized cumulant density responses in both muscles (mGAS: λ^2^ = 18.375, *p* < 0.001; SOL: λ^2^ = 13.625, *p* = 0.001). Pairwise analysis revealed that normalized cumulant density responses in both muscles were largest for the 1F condition and decreased by ∼27–33% when load was increased to 1.5F (mGAS: *Z* = −3.516, *p* = 0.001; SOL: *Z* = −3.361, *p* = 0.002) (Figure 4D). When the load was increased further to 2F, however, responses were similar to those observed in the 1.5F condition (mGAS: *Z* = −0.414, *p* = 0.679; SOL: *Z* = −1.086, *p* = 0.278), and decreased only relative to the normal load condition (mGAS: *Z* = −3.309, *p* = 0.002; SOL: *Z* = −3.154, *p* = 0.005). Non-normalized cumulant density responses, in contrast, showed a progressive increase with additional load (see Figure 4B insets) in both the gastrocnemius (47 and 74%, respectively, λ^2^ = 17.375, *p* < 0.001) and soleus muscles (7 and 50%, respectively, λ^2^ = 19.500, *p* < 0.001). Our results also indicated that the timing of the short and medium latency peaks in both muscles were effected by additional load (short latency: mGAS – λ^2^ = 12.132, *p* = 0.002, SOL – 12.984, *p* < 0.002; medium latency: mGAS – λ^2^ = 26.000, *p* < 0.001, SOL – λ^2^ = 17.322, *p* < 0.001) (see Table 2). Pairwise analysis indicated that with elevated load (1.5 and 2F), the short latency peaks occurred ∼1.4–2.4 ms earlier (mGAS: multiple *p* < 0.002; SOL: multiple *p* < 0.006), and the medium latency peaks occurred ∼2.0–4.9 ms earlier (mGAS: multiple *p* < 0.002; SOL: multiple *p* < 0.04) compared to the normal 1F load condition (see Table 2). Similar to the normalized peak-to-peak amplitudes, we found no differences between the timing of the short and medium latency peaks across the 1.5F and 2F conditions for both muscles (mGAS: multiple *p* = 1.0; SOL: multiple *p* = 1.0).

**Table 2:** Group data of peak-to-peak normalized and non-normalized cumulant density responses responses, as well as timing of the short- and medium latency responses.

		Normalized cumlant density peak-to-peak [-]		Non-normalized cumulant density peak-to-peak [uV mA]		Short latency [msec]		Medium latency [msec]	
					
		Gas	Sol	Gas	Sol	mGAS	SOL	mGAS	SOL
		*median / IQR*	*median / IQR*	*median / IQR*	*median / IQR*	*median / IQR*	*median / IQR*	*median / IQR*	*median / IQR*
**Exp 1**	*1F*	0.19/0.10	0.11/0.05	11.41/10.12	6.62/5.92	61.3/2.8	62.3/3.8	103.3/3.8	101.8/5.0
	*1.5F*	0.14/0.10	0.07/0.05	16.81/21.70	6.97/7.59	59.5/4.5	59.8/4.4	98.5/5.0	99.3/4.9
	*2F*	0.15/0.10	0.07/0.05	19.87/24.99	9.54/9.48	59.8/3.0	58.8/3.5	97.8/4.5	100.0/3.9
**Exp 2A**	*1F*	0.16/0.08	0.10/0.09	8.65/18.48	5.64/9.18	61.9/7.9	62.8/6.9	107.3/18.5	114.8/19.5
	*2F*	0.13/0.09	0.06/0.08	13.98/17.72	7.17/14.43	60.8/8.6	63.0/5.6	98.3/14.8	103.3/15.4
**Exp 2B**	*0G-1F*	0.12/0.05	0.09/0.06	6.94/5.25	7.85/5.37	62.5/7.8	66.5/5.5	108.5/12.0	113.5/11.5
	*1G-1F*	0.17/0.12	0.12/0.10	15.68/11.66	10.97/9.22	60.8/2.1	62.5/5.0	107.8/6.4	109.5/7.5
	*1.8G-1.8F*	0.12/0.07	0.11/0.05	23.21/13.41	15.65/12.09	57.3/5.3	61.0/3.5	99.3/7.5	102.0/9.5
	*1G-2F*	0.16/0.06	0.09/0.04	21.20/14.29	12.40/6.46	60.3/7.4	61.5/4.0	97.8/13.4	104.5/7.1

*mGAS, medial gastrocnemius muscle; SOL, soleus muscle.*

The six subjects who participated in the on-ground training (Experiment 2A) showed similar patterns of balancing behavior and vestibular-evoked muscle responses with changing load levels as seen in Experiment 1 (see Table 1). Under increased load (2F), muscle activity (mGAS: 89.6%, *Z* = −2.201, *p* = 0.028; SOL: 71.6 %, *Z* = −2.201, *p* = 0.028) and ankle torque (114.6%, *Z* = −2.201, *p* = 0.028) increased substantially relative to 1F loading. Furthermore, vertical load forces in the 2F condition (185.6%) were slightly below the intended level of 2 times the subjects’ body weight (Table 1). Minor differences relative to Experiment 1, however, were observed; mean whole-body sway angle increased by 3% (*Z* = −1.992, *p* = 0.046) during 2F loading, while RMS sway was similar across conditions (*Z* = −1.153, *p* = 0.249). Nevertheless, the vestibular-evoked muscle responses in these six subjects also showed similar changes with increasing load when compared to those observed in Experiment 1. During 2F loading, DoC tests revealed that pooled coherence in both muscles decreased between 0 and ∼8 Hz (data not shown). Similarly, in both muscles, the normalized cumulant density responses decreased during 2F loading (mGAS: 42%, *Z* = −2.201, *p* = 0.028; SOL: 19%, *Z* = −2.201, *p* = 0.028), while the non-normalized cumulant density responses increased (mGAS: 62%, *Z* = −2.201, *p* = 0.028; SOL: 27%, *Z* = −2.201, *p* = 0.028) (see Table 2). Finally, timing of the short- and medium latency peaks with additional load occurred ∼1.3–2.5 ms (mGAS*: Z* = −2.201, *p* = 0.028) and ∼6.4–7.5 ms earlier (mGAS: *Z* = −2.201, *p* = 0.028; SOL: *Z* = −2.207, *p* = 0.027) relative to normal standing, respectively, with the exception of the soleus short latency peak which did not differ across conditions (SOL*: Z* = −0.949, *p* = 0.343).

Overall, the results of Experiments 1 and 2A indicate that although non-normalized vestibular-evoked muscle responses increase with additional load, the relative vestibular contribution (i.e., normalized cumulant density) is reduced and slightly advanced compared to normal balance conditions. However, no further changes in the amplitude and timing of the relative vestibular contribution are observed when the load exceeds 1.5 times the body weight.

### Effect of Vestibular Cues of Gravity on Vestibular-Evoked Muscle Responses (Experiment 2B)

When subjects balanced during the in-flight experiments (i.e., Experiment 2B), we observed an increased difficulty in maintaining upright stance. Plane turbulence and unexpected loads throughout the parabola caused some subjects to fall into the end stops. As a result, the mean-removed RMS whole-body sway was ∼2–3 times higher during in-flight testing as compared to on-ground training (Table 1). Nevertheless, the general balance behavior from Experiment 2B showed similar trends as Experiment 2A when the total load was increased (i.e., 0G-1F/1G-1F vs. 1.8G-1.8F/1G-2F). Specifically, mean foot loading, ankle torque and muscle activity increased under conditions with higher load. Variations in balance behaviors, however, were observed when comparing responses across the different gravity levels as detailed below.

#### Comparison of 0G-1F and 1G-1F Conditions

**FIGURE 5:**
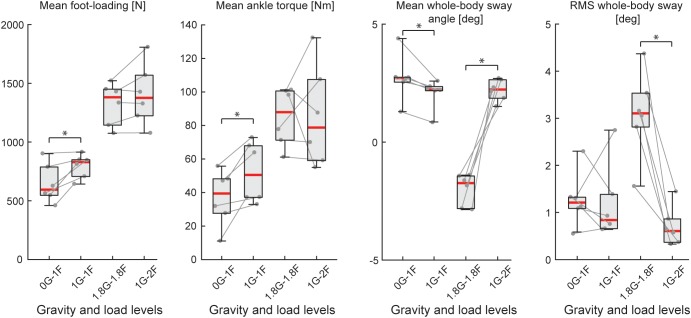
Outcome measures of general balance behavior during Experiment 2B (*n* = 6). Responses include vertical loading forces, ankle torque and whole-body sway angle (mean and mean-removed RMS). Individual subjects are plotted as gray dots. Data are plotted as median (red line), 25 and 75 percentiles (gray box) and extreme data points (gray whiskers). ^∗^*p* < 0.05 indicates significant differences between conditions.

**FIGURE 6:**
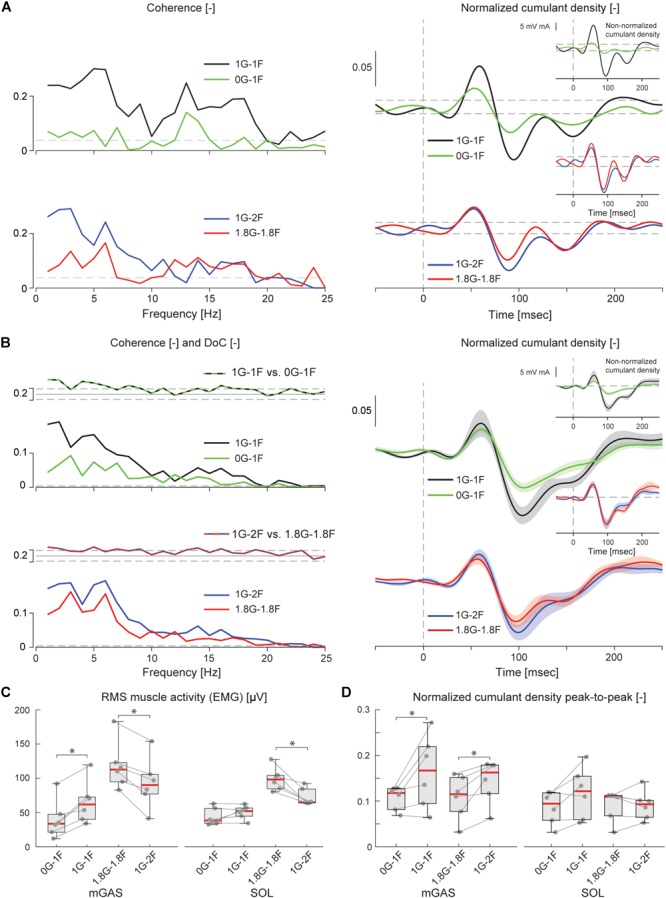
Vestibular-evoked muscle responses with varying load and gravity levels from Experiment 2B. Data from the medial gastrocnemius muscle are shown for a single subject **(A)** and group responses (*n* = 6) **(B)**. In general, variations in gravity and load level relative to normal standing conditions resulted in lowered coherence and cumulant density responses. Horizontal dotted lines indicate the 95% confidence limits for coherence, difference-of-coherence and cumulant density responses. DoC results are plotted above the group pooled coherence results. A positive value for the DoC indicates greater coherence for the first condition listed in each comparison; whereas a negative value represents greater coherence for the second condition in each comparison. Cumulant density plots show the normalized and non-normalized (insets) responses for both individual subject and group data. In the group cumulant density responses, bold lines are group means and for illustrative purposes shaded areas show the standard error. For comparison, data from the group (*n* = 6) root-mean-square of muscle activity (EMG) **(C)** and peak-to-peak amplitudes of the normalized cumulant density responses **(D)** are shown. Individual subjects are plotted as gray dots. Group data are plotted as median (red line), 25 and 75 percentiles (gray box) and extreme data points (whiskers). ^∗^*p* < 0.05 indicates significant differences between conditions.

During the micro-g phase of the parabola (i.e., 0G-1F condition), all subjects were able to maintain upright balance without difficulty. Load forces during the 0G-1F condition, however, were 18.6% lower than the 1G-1F conditions (*Z* = −2.201, *p* = 0.028) (see Figure 5). As a result, ankle torque (*Z* = −2.201, *p* = 0.028) and medial gastrocnemius muscle activity (*Z* = −2.201, *p* = 0.028) were also 31.9 and 38.6% lower during the micro-g condition, respectively. Notably, however, there was no difference in the soleus muscle activity across gravitational conditions (*Z* = −0.943, *p* = 0.345) (see Figure 6C). Finally, mean whole-body sway angle during the micro-g condition was larger relative to the normal 1G-1F condition (*Z* = −2.201, *p* = 0.028) – though this was attributed to a single subject who leaned too far forward – while mean-removed RMS sway was not significantly different (*Z* = −0.734, *p* = 0.463) (Table 1 and Figure 5).

The electrical stimulus evoked significant muscle responses in all subjects during trials both with and without gravity. Data from a representative subject show that during the micro-g condition, both coherence and cumulant density responses in the medial gastrocnemius muscle decreased relative to the normal condition (Figure 6A black and green traces). Similar responses were observed in the group data (Figure 6B black and green traces): the DoC test indicated that pooled coherence decreased when subjects stood without gravity at frequencies between 0 and ∼10 Hz, but only in the medial gastrocnemius muscle (see Figure 6A black and green traces; note SOL muscle data not shown). This decrease in medial gastrocnemius coherence was associated with a 30% decrease in the normalized cumulant density (*Z* = −1.992, *p* = 0.046) (Figure 6D), and was accompanied by a 29% decrease in the non-normalized cumulant density (*Z* = −2.201, *p* = 0.028) (Figure 6B inset, black and green traces). In the soleus muscle, a similar decreasing trend in the responses of both cumulant density measures was observed during micro-g conditions, however, these differences were not significant (normalized: *Z* = −1.782, *p* = 0.075; non-normalized: *Z* = −1.363, *p* = 0.173) (Figure 6D). Furthermore, we also found no change in the timing of the short and medium latency peaks across gravity conditions for both the medial gastrocnemius (short: *Z* = −0.535, *p* = 0.593; medium: *Z* = −0.135, *p* = 0.892) and soleus muscle (short: *Z* = −0.184, *p* = 0.854; medium: *Z* = −0.674, *p* = 0.500) (see Table 2).

#### Comparison of 1.8G-1.8F and 1G-2F Conditions

Throughout the hyper-g phase of the parabola (i.e., 1.8G-1.8F condition), plane accelerations in the direction of balance (i.e., longitudinal axis of the plane) progressively increased and tended to push the subjects forward. This additional load made it difficult for subjects to maintain the desired whole-body sway angle without falling into the forward end stop. Consequently, the subjects were instructed to stand leaning forward at an angle that required similar effort as the condition when additional load was provided by the springs with normal 1 g gravity (i.e., 1G-2F). This ensured that the muscles were engaged in a task to remain upright but required that subjects stand at a mean whole-body sway angle that was ∼4° anterior relative to the 1G-2F condition (*Z* = −2.201, *p* = 0.028). Despite this difference in sway angle, load forces and ankle torque were similar across normal and hyper-g conditions (i.e., 1.8G-1.8F vs. 1G-2F, see Table 1 and Figure 5; load forces: *Z* = −1.153, *p* = 0.249; ankle torque: *Z* = −0.314, *p* = 0.753). However, muscle activity was ∼21–27% higher during hyper-g trials (mGAS: *Z* = −2.201, *p* = 0.028; SOL: *Z* = −2.201, *p* = 0.028) and the mean-removed RMS sway was about three times higher (*Z* = −2.201, *p* = 0.028). These differences in muscle activity and sway variability were likely due to the variation in longitudinal acceleration that occurred during the hyper-g phase.

Despite the differences in general balance behavior, the electrical stimuli evoked significant muscle responses in conditions with and without the additional gravity. Data from a representative subject show that during the hyper-g condition, coherence and normalized cumulant density responses in the medial gastrocnemius muscle decreased relative to the normal condition (Figure 6A red and blue traces). The non-normalized cumulant density response, however, showed no obvious variation in response magnitude across conditions (Figure 6A inset, red and blue traces). A similar effect of the additional gravity was observed in the group data: the DoC test showed coherence decreased during hyper-g trials at most frequencies between 0 and ∼10 Hz, but only in the medial gastrocnemius muscle (Figure 6B red and blue traces; note SOL muscle data not shown). Further, this decrease in medial gastrocnemius coherence was associated with a 29% decrease in the normalized cumulant density (*Z* = −2.201, *p* = 0.028), but no significant difference in the non-normalized cumulant density (*Z* = −0.105, *p* = 0.917) (see Figure 6B inset, red and blue traces). Soleus muscle responses, in contrast, showed no significant difference with the additional gravity for both the normalized and non-normalized cumulant density responses (both: *Z* = −0.105, *p* = 0.917) (see Table 2). Similar to the micro-g conditions, timing of the short and medium latency peaks showed no change between conditions for both the medial gastrocnemius (short: *Z* = −1.604, *p* = 0.109; medium: *Z* = −0.943, *p* = 0.345) and soleus muscle (short: *Z* = −1.095, *p* = 0.273; medium: *Z* = −1.625, *p* = 0.104) (see Table 2).

Overall, the results of Experiment 2B indicate that vestibular input to muscle activity persist across varying levels of gravity, but that the relative contribution of vestibular input to ongoing muscle activity decreases when vestibular cues of gravity decrease (but perhaps not increase) relative to normal 1 g gravity.

## Discussion

The aim of the present study was to determine whether both somatosensory and vestibular cues of gravity modify the corrective muscle actions to vestibular-evoked postural disturbances. Our results show that when subjects balanced with added load and a constant 1 g vestibular signal, the relative vestibular contribution to the evoked muscle responses (i.e., coherence and normalized cumulant density) decreased and occurred earlier relative to responses during normal standing. In addition, when subjects balanced with varying levels of gravity while the overall load was held relatively constant, the relative vestibular contribution to evoked muscle responses also decreased. This modulation, however, was primarily limited to micro-g conditions when vestibular cues of gravity were absent. Furthermore, these response reductions with changes in gravity occurred in absence of any significant change in their timing. These results demonstrate that load-related cues of gravity from the somatosensory system (i.e., cutaneous and proprioception) and vestibular-related cues of gravity from the otolith end organs influence the vestibular drive for standing balance, such that the relative vestibular contribution to corrective postural responses decreases when sensory cues of gravity differ from normal 1 g conditions.

Despite the reduction in the normalized vestibular-evoked responses across varying load and gravity levels, significant muscle responses to the electrical stimulus were observed for all subjects in all conditions. This aligns with the notion that vestibular-evoked muscle corrections during quiet standing are only evoked when both vestibular information and a muscle’s contribution are relevant to the process of balancing the body (Britton et al., 1993; Fitzpatrick et al., 1994; Luu et al., 2012; Forbes et al., 2016). For instance, responses are absent when standing subjects balance a body-equivalent inverted pendulum while being supported by a rigid backboard, a condition where somatosensory signals – but not vestibular signals – are relevant to the balance task (Fitzpatrick et al., 1994). Therefore, it is not entirely surprising that vestibular-evoked muscle responses were observed across our changing load and gravity conditions since both the vestibular feedback and the muscle corrections were always engaged in, and/or relevant to, balancing the body against a downward pulling force. Under micro-gravity conditions, the otolith sensory cues produced by gravity were removed, limiting the available sensory cues of the downward pulling force to somatosensors only. Our results therefore demonstrate that vestibular contributions to standing can be maintained with sensory feedback signals of load and balance that are absent of the otolithic signal of gravity (e.g., somatosensory and/or dynamic vestibular signals). Accordingly, it may be possible that even after prolonged exposure to micro-gravity in space-flight, vestibular-evoked muscle responses continue to compensate for vestibular disturbances while balancing the body against a downward load, in contrast to the reduced otolith-spinal reflexes during the specific freefall drop conditions (Reschke et al., 1984, 1986; Watt et al., 1986). The sustained influence of a vestibular signal for balance in the absence of gravity also parallels the observation that when balancing without proprioceptive signals of ankle angle (i.e., sway referenced balance) (Nashner and Wolfson, 1974; Luu et al., 2012; Forbes et al., 2016) or visual signals of body sway (i.e., in the dark or with eyes closed) (Fitzpatrick et al., 1996; Welgampola and Colebatch, 2001) vestibular-evoked muscle responses are retained.

The changes in vestibular-evoked responses observed here also align with the influence that varying sensory cues of standing can have on the vestibular control of balance (Nashner and Wolfson, 1974; Lund and Broberg, 1983; Britton et al., 1993; Welgampola and Colebatch, 2001; Muise et al., 2012). Cooling of the feet, for example, reduces the sensitivity of cutaneous receptors and increases vestibular-evoked muscle responses (Muise et al., 2012). Similarly, additional load on the body decreases cutaneous receptor sensitivity (Mildren et al., 2016), and progressively increases the associated vestibular-evoked postural responses (Marsden et al., 2003). At first glance, our results when increasing the load (Experiments 1 and 2A) seem to contradict the study of Marsden et al. (2003), since the normalized vestibular-evoked muscle responses reported here (a) decreased with additional load, and (b) ceased to vary (or plateau) when the load was increased from 1.5 to 2 times the body weight. Marsden et al. (2003) however, examined the rate of reaction force development evoked by a transient electrical stimulus, a non-normalized response that reflects the net contribution of vestibular input to postural control. Indeed, similar to Marsden et al. (2003) we found that the non-normalized cumulant density responses also increased with additional load. Our normalized cumulant density results therefore extend the findings of Marsden et al. (2003) showing that although the total vestibular contribution progressively increases with the excitability of the motoneuron pool at higher loads, the relative contribution of vestibular signals decreases. As the load increases beyond 1.5 times body weight, however, the relative vestibular input remains constant.

Our results from Experiment 2B further demonstrate that a decreasing vestibular cue of gravity also influences the processing of vestibular information for balance. Under micro-g conditions, normalized vestibular-evoked muscle responses decreased relative to standing with 1 g vestibular cues and matching load cues (1F). The covariation of both the normalized and non-normalized cumulant density responses together with EMG magnitude (i.e., all measures decreasing) during micro-g trials suggests that the net decrease in the input to the motoneuron was accompanied by a proportionally larger decrease in the vestibular contribution. During hyper-g conditions, in contrast, we saw a reduction only in the normalized cumulant density together with an increase in EMG magnitudes. It appears likely that the decrease in the normalized cumulant density responses during hyper-g was simply due to a net increase in the input to motoneuron arising from non-vestibular sources (Bacsi and Colebatch, 2005; Heroux et al., 2015). A confounding factor in interpreting these hyper-g results, however, is that the loading conditions were substantially different across the two conditions. In particular, the mean sway angle during hyper-g trials was ∼4 degrees posterior and the RMS sway angle was three times higher relative to the 1 g (i.e., 1G-2F) condition. Accordingly, we cannot rule out the possibility that variations in balance state across conditions (i.e., sway angle, sway velocity) (Lee Son et al., 2008; Forbes et al., 2018; Rasman et al., 2018) also contributed to any effect caused by changing gravity. Finally, an additional limitation to these results is that the observed changes in vestibular contributions across gravity conditions in the medial gastrocnemius muscles were not observed in the soleus muscle. However, given the reduced sensitivity of the soleus muscle to vestibular input as compared to the medial gastrocnemius muscle (Dakin et al., 2016), it may be possible that an effect could be seen if the number of subjects was increased.

Overall, the results of both experiments indicate that changes in load and vestibular cues of gravity primarily decrease the relative contribution of vestibular signals to ongoing muscle activity. This reduction in the normalized vestibular-evoked muscle responses with changes in multiple sensory cues of gravity may be compatible with previous observations that the amplitude of vestibular-induced balance responses are dependent upon the congruency between actual and expected sensory consequences of postural motor actions (Luu et al., 2012). The balance system is thought to predict the sensory consequences of postural tasks using an internal model of the standing body’s dynamics under normal 1 g loading (van der Kooij et al., 2001; Kuo, 2005; Heroux et al., 2015; Forbes et al., 2018). When the sensory predictions produced by the internal model do not match actual sensory feedback, vestibular input to standing balance decreases. Therefore, when subjects balance with altered load or vestibular cues of gravity, the change in congruent sensory signals relative to normally expected 1 g standing produces similar changes (i.e., reductions) in the vestibular-evoked motor responses. Importantly, this does not exclude the possibility for adaptation to any of these altered sensory conditions, which over sufficient exposure may allow the vestibular-evoked responses to return to expected levels (Heroux et al., 2015).

## Conclusion

The present study shows that the vestibular drive for standing balance was always present across variations in load- and vestibular-related cues of gravity, but that the relative vestibular contribution was attenuated when these signals were altered from normal 1 g conditions. This suggests that multiple afferent feedback cues of gravity influence the contribution of vestibular signals for the control of upright stance. Our study provides unique insight into the effect that changing levels of gravity can have on the sensorimotor processing for standing balance and may have important implications for astronauts interacting in different levels of gravity.

## Ethics Statement

The study protocol was approved by the Medical Research Ethics Committee Erasmus MC (Experiment 1) and the University of Caen’s Ethics Committee (Experiment 2). The experiments were conducted in accordance with the Declaration of Helsinki and all subjects gave their written informed consent prior to participation.

## Author Contributions

AA and PF contributed to the conception and design of the study, analyzed the data and wrote the first draft of the manuscript. ZJ created the custom MATLAB software. AA, DvP, CH, and PF collected the data. All authors contributed to manuscript revisions, and read and approved the submitted version.

## Conflict of Interest Statement

The authors declare that the research was conducted in the absence of any commercial or financial relationships that could be construed as a potential conflict of interest.
